# Transcription Factors: The Fulcrum Between Cell Development and Carcinogenesis

**DOI:** 10.3389/fonc.2021.681377

**Published:** 2021-06-14

**Authors:** Zeyaul Islam, Ameena Mohamed Ali, Adviti Naik, Mohamed Eldaw, Julie Decock, Prasanna R. Kolatkar

**Affiliations:** ^1^ Diabetes Center, Qatar Biomedical Research Institute (QBRI), Hamad Bin Khalifa University (HBKU), Qatar Foundation, Doha, Qatar; ^2^ Translational Cancer and Immunity Center, Qatar Biomedical Research Institute (QBRI), Hamad Bin Khalifa University (HBKU), Qatar Foundation, Doha, Qatar

**Keywords:** transcription factors, cell fate, pluripotency, tumorigenesis, cancer mechanisms, clinical relevance

## Abstract

Higher eukaryotic development is a complex and tightly regulated process, whereby transcription factors (TFs) play a key role in controlling the gene regulatory networks. Dysregulation of these regulatory networks has also been associated with carcinogenesis. Transcription factors are key enablers of cancer stemness, which support the maintenance and function of cancer stem cells that are believed to act as seeds for cancer initiation, progression and metastasis, and treatment resistance. One key area of research is to understand how these factors interact and collaborate to define cellular fate during embryogenesis as well as during tumor development. This review focuses on understanding the role of TFs in cell development and cancer. The molecular mechanisms of cell fate decision are of key importance in efforts towards developing better protocols for directed differentiation of cells in research and medicine. We also discuss the dysregulation of TFs and their role in cancer progression and metastasis, exploring TF networks as direct or indirect targets for therapeutic intervention, as well as specific TFs’ potential as biomarkers for predicting and monitoring treatment responses.

## Introduction

To establish and maintain specific cell lineage during development, a complex and tightly regulated gene expression network is active under the control of both intrinsic and extrinsic signaling pathways that culminate in the activation of transcription factors (1). Transcription factors (TFs) play a major role in regulating gene expression by recognizing and directly binding to specific DNA sequences. This binding then results in direct and/or indirect transcription activation of downstream genes, bringing RNA polymerase and other transcriptional machinery to the promoter sequence (2–4). TFs can also regulate expression through the recruitment of corepressors or by interfering with the binding of other TFs (5, 6).

During the early stages of embryo development, asymmetrical cell divisions along a basolateral cleavage plane create inner cell mass (ICM) and outer cell mass of trophectoderm (TE) lineage (7, 8). This hallmark event in early mammalian development is mainly dictated by two transcription factors; the POU-family transcription factor octamer-binding transcription factor 3/4 (Oct3/4) and the caudal-type homeobox protein 2 (Cdx2). Oct3/4 and Cdx2 establish a mutually exclusive expression pattern and form a complex for reciprocal repression of their target genes, suggesting reciprocal inhibition between lineage specific TFs during early stages of mammalian differentiation and development where Cdx2 drives lineage towards trophectoderm (9, 10). In addition, the Tea-domain family member 4 (Tead4) transcription factor regulates TE lineage specification (11, 12) through differential subcellular regulation (11). Furthermore, the early ICM contains a cell subpopulation that leads to the formation of two lineages, the epiblast (EPI) and primitive endoderm (PE), which are differentially regulated by Nanog (a homeobox TF in EPI) or Gata6 (Gata binding factor 6 in PE). Nanog and Gata6 are expressed in the ICM in an apparently random and mutually exclusive manner (13, 14). Nanog, together with Sall4, is vital to maintain the self-renewal capability of the ICM and of embryonic stem (ES) cells, derived from the inner cell mass (ICM) (15–18). Sall4 regulates Oct4 by binding to its conserved region, thus availing it for maintenance of ES cell pluripotency (18, 19) while Sall1 is implicated in the development of kidney, heart, and other systems (20). ES cells can maintain pluripotency and generate somatic and germ cell lineages of the developing embryo (17), and ESC pluripotency is governed by the core transcription factors Nanog, Oct4, Klf4, and Sox2 among others (17). Other key developmental transcription factors include the FoxA, Pax, and Ppar**γ** families. FoxA family proteins are well known early developmental transcription factors (21) and are hence also known as “pioneer factors”. The Pax family TFs are involved in maintenance of progenitor cells across a wide variety of tissues including the thymus, pancreas and eye (20). Ppar**γ** proteins are involved in many functions including cell proliferation and development of several tissues, and gliomas (20).

Given the vital role of TFs in determining cell fate, the extrapolation to tumor development and progression is easily made. Epithelial to mesenchymal transition (EMT) is not only an essential embryonic process during which apical-basal polarized epithelial cells convert into motile front-back polarized mesenchymal cells, but it is also crucial for ‘tissue invasion and metastasis’, a hallmark of cancer. The plasticity of cancer cells to switch between an epithelial and mesenchymal phenotype bestows them with the ability to survive the hostile tumor microenvironment and to colonize distant organs. Multiple theories exist on the identity of cancer cells with such abilities, supporting either the presence of a specific subpopulation of cancer stem cells (CSCs) within the bulk tumor or a subset of cancer cells with high plasticity, or a combination of both theories. Irrespective of the theories on CSC origin, cancer cells with stemness features are associated with the ability to self-renew and propagate unlimited. Less-differentiated tumors contain higher amount of CSCs as compared to well-differentiated tumors (22). Moreover, CSCs have been involved in tumor initiation, metastasis, and resistance to chemotherapy and radiotherapy (22, 23). The expression profiles of TFs involved in CSCs maintenance are similar to what is found in ESCs as compared to what is observed in adult stem cells (24, 25). The aim of this review is to summarize the current knowledge and highlight differences in the role of transcription factors that are involved in cell fate control during normal tissue as well as tumor development. Transcription factors involved in early as well as key developmental stages and those with strong cancer links were specifically chosen for this review. This review also tries to give a wider breadth of different types of TFs to better capture the diversity of involved TFs rather than focus on any single family of TFs or type of cancer.

## Forkhead Box A

The Fox family encompasses more than 170 transcription factors with a conserved winged-helix DNA-binding domain (DBD) (26–28). These proteins participate in cellular processes ranging from development to immunity and metabolism (26, 27, 29–31). The Fox family can be stratified into 19 subfamilies, FoxA to FoxS, based on protein sequence homology (32). Fox proteins share a signature 80–100 amino acid DNA-binding domain known as forkhead box but significantly differ in other regions, allowing for differential expression, regulation, and functional diversification (29, 33).

The FoxA subfamily, known as hepatocyte nuclear factor 3 (Hnf3), comprises three members, FoxA1, FoxA2, and FoxA3, that can remodel nucleosomes and facilitate DNA binding of other TFs (21, 34). FOXA members have been depicted as pioneer factors because of their ability to bind transcription factor binding sites (Tfbs) located in condensed, inactive chromatin in order to initiate chromatin remodeling and support other TFs in accessing chromatin to prompt their tissue-specific functions such as estrogen and androgen modulation (21, 34–36).

**Figure 1:**
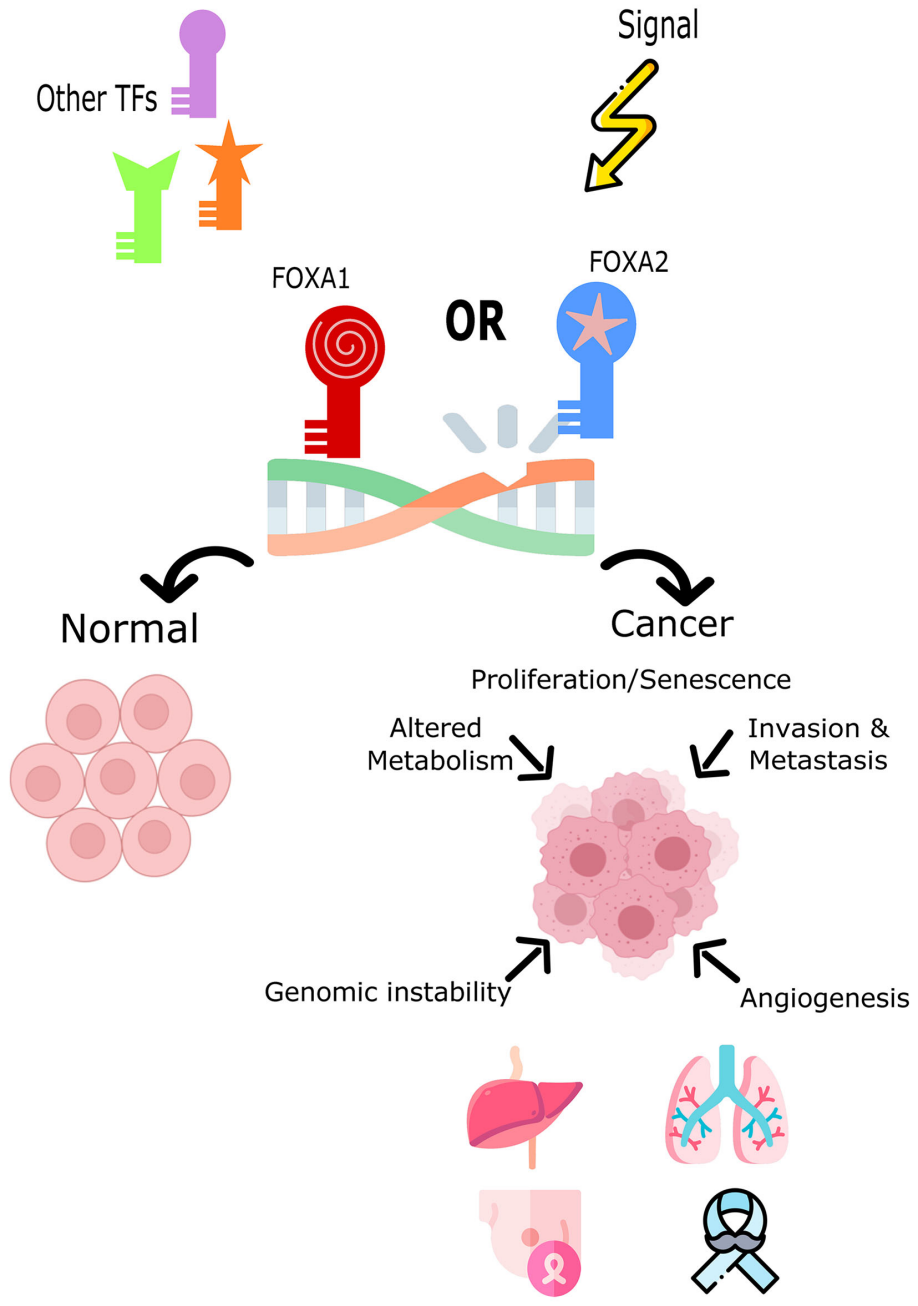
The role of FoxA1 and FoxA2 in cell fate decision or tumor induction. Both TFs impact the cell’s development toward normal cycle and differentiation or toward cancer and tumorigenesis. FOXA1 and FOXA2 overexpression, mutation, or down-regulation is associated with different cancers such as, lung, liver, breast, and prostate cancers.

Increased expression of Fox proteins has been observed in a wide range of cancers and is commonly associated with advanced cancer stages and poor survival *via* increased proliferation (37). Several studies have demonstrated a role for FoxA1 and FoxA2 in the regulation of cell cycle progression, proliferation and differentiation, genomic instability and DNA damage repair, metabolism, angiogenesis, invasion, and senescence (**Figure 1**). In comparison, sparse data is available on the role of FoxA3 in cancer. One recent study demonstrated an increase in FoxA3 expression in esophageal cancer, which was associated with increased invasion, distant metastasis, disease stage, and a shorter overall survival (38). However, these TFs are correlated with oncogenic but also conversely tumor-suppressive functions (inhibiting metastasis) depending on how they interact with the transcriptional networks of tissue-specific cancers (29, 39, 40). **Table 1** summarizes the expression patterns of FoxA protein in various cancers.

**Table 1:** The association between each member of FoxA family and different cancers.

FoxA member	Expression	Cancer type	Reference
**FoxA 1**	Increased	Lung cancer	(41, 42)
		Breast cancer	(41, 43–48)
		Prostate cancer	(45, 49–56) (57) (58)
		Liver cancer	(59)
		Breast cancer	(57)
		Gastric cancer	(60)
		Ovarian cancer	(61)
		Esophageal cancer	(41)
		Thyroid cancer	(62)
	Mutation	Invasive lobular carcinoma (ILC)	(63)
**FoxA 2**	Increased	Liver cancer	(59)
		Prostate Cancer	(64, 65)
		Hepatocarcinoma	(66)
		Breast cancer	(47)
	Decreased	Pancreatic cancer	(67)
		“Pancreatic ductal adenocarcinoma (PDAC)”	
		Bladder cancer	(68, 69)
		“Muscle-invasive bladder cancer”	

## OCT4

**Figure 2:**
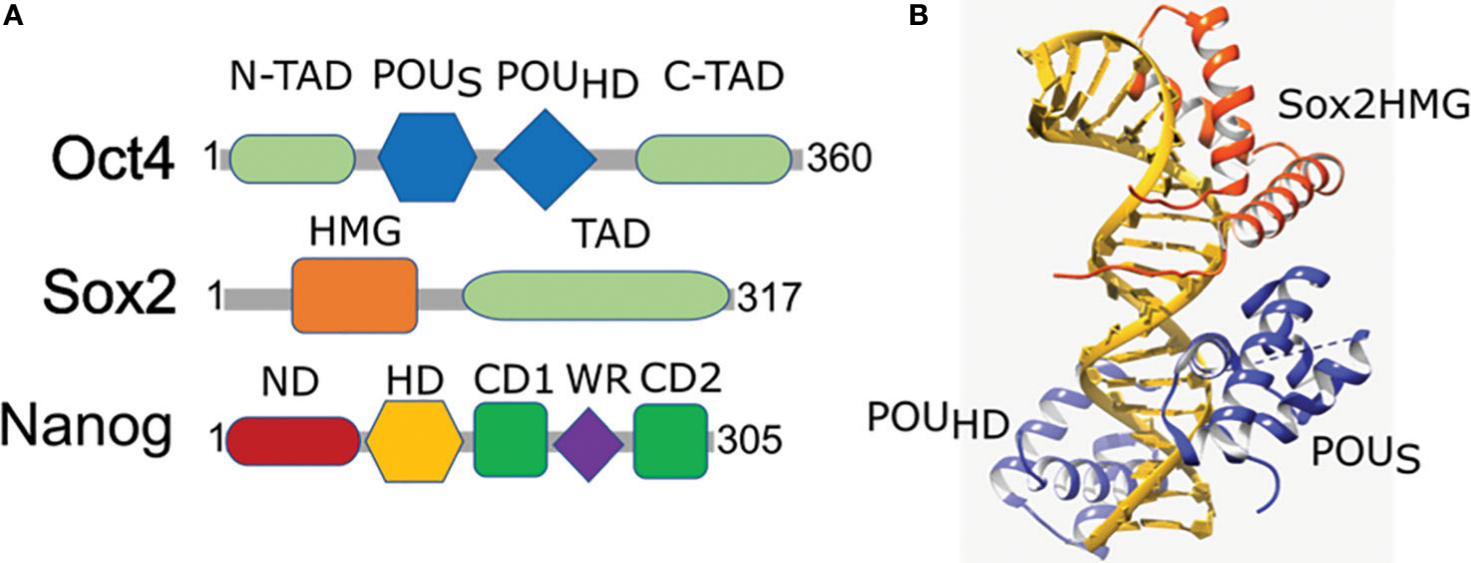
Domain organization and structural arrangement of Oct4, Sox2, and Nanog. **(A)** Oct4 has DNA-binding domains (a POU-specific DNA-binding domain (POU_S_) and a POU-homeodomain (POU_HD_)) interacting independently with DNA as well as transactivation domains located N-terminal (N-TAD) and C-terminal (C-TAD). Sox2 is a High mobility group (HMG) family member and has a single HMG DNA-binding domain and a transactivation domain (TAD). Nanog has N-terminal containing a DNA-binding homeodomain (HD) and an N-terminal domain (ND), C-terminal containing a dimerization domain (blue) referred to as the tryptophan repeat (WR), that separates C-terminal domain 1 (CD1) from C-terminal domain 2 (CD2). **(B)** Ternary structure of Oct–Sox–DNA (PDBID: 1O4X). Sox2 binding to DNA and Oct4 is enabled by the HMG domain (orange) that cooperates in binding of Oct4 POU domain (blue) onto the DNA (golden).

Oct4, also known as Pou5f1, is one of the core transcription factors that regulates ESC pluripotency (80). It contains three domains; the DNA binding POU domain, C-terminal transactivation domain, and variable N-terminal domain and binds an octamer sequence motif (ATGCAAAT) to regulate the expression of its target genes (**Figure 2A**) (81, 82). The expression level of Oct4 is critical for ESCs’ fate determination whereby an intermediary level maintains the self-renewal capacity of ESCs, a decrease in the expression level supports differentiation into trophectoderm lineage, and a subsequent increase induces differentiation into primitive endoderm or mesoderm (83).

Oct4 expression has been observed in numerous cancers, with increased expression in more aggressive tumors and decreased expression being associated with regression of tumor potential (84). For instance, high expression of Oct4 in combination with other core pluripotency factors has been linked with pancreatic carcinogenesis, whereas silencing of Oct4 results in decreased proliferation, migration, invasion, and chemoresistance (85). Accordingly, multiple studies have demonstrated a correlation between Oct4 expression and treatment resistance and poor survival. For example, Oct4 expression is increased in docetaxel and mitoxantrone-resistant prostate cancer, cisplatin-resistant lung and ovarian cancer, radiation-resistant cervical cancer and chemo-resistant oral squamous carcinoma cancers (82, 86, 87). Conversely, Oct4-knockdown has been shown to increase the sensitivity to cisplatin treatment and radiotherapy in lung and ovarian cancers and to temozolomide in glioma-initiating cells (88–91). In contrast, testicular germ cell tumors display an inverse relation between Oct4 expression and resistance to cisplatin, although the exact mechanism is not yet clear (92, 93). Although increased Oct4 is generally associated with better outcome, in these tumors Oct4 expression was correlated with worse survival, similar to what has been observed in hypopharyngeal squamous cell carcinoma (94). This discrepancy in prognostic connotation underlines the biological complexity of this TF in cancer biology. In analogy with most stem cell factors, the level of Oct4 expression appears to be critical in cancer whereby either increased or decreased expression can perturb distinct cancer-related pathways.

## SOX2

Sox2 is well established as a key transcription factor for self-renewal and pluripotency of neural stem cells and undifferentiated ESCs and is an integral part of embryogenesis, organogenesis, and overall animal development (95–98). It is a member of the Sox family of proteins that contain a DNA-binding high mobility group (HMG) domain that forms a concave surface enabling binding to DNA in a sequence-specific manner (99–101). The subsequent conformational change then unwinds the DNA, which helps to recruit other TFs, coactivators, or repressors (**Figure 2B**). The differential partnership of a functional Sox2 DNA binding site in combination with a second binding site for a partner protein determines the overall transcriptional activation or repression (102). Furthermore, Sox2 and Oct4 co-binding is required for gene activation at several loci providing more support that Sox2 and its partner factors are recruited to unique target sequences in specific conformations for transcriptional regulation (102). As such, partner switching plays an important role in differential gene expression. For example, during endodermal differentiation, the Sox2–Oct4 complex switches into a Sox17–Oct4 complex (103) as a result of Sox2 and Sox17 competing for binding to Oct4 and forming stable complexes on specific regions to determine the cell fate (104).

Of note, Sox2 is considered a proto-oncogene whereby Sox2 gene amplification, mutation, and overexpression can lead to multiple malignant conditions with metastasis (105–109). Sox2 amplification is positively correlated with increased proliferation, tumor burden metastasis, and poor prognosis (110–114). In tongue squamous cell carcinoma, Sox2 overexpression was associated with EMT progression, suggesting its involvement in regulation of cancer cell motility (115). Furthermore, Sox2 has been associated with tumorigenicity, illustrating its role in cancer stemness. For example, overexpression of Sox2 speeds up tumorigenicity in I-type neuroblastoma cells (116). Conversely, Sox2 silencing reduced tumorigenicity of glioblastoma and lung cancer tumor initiating cells (117, 118) and of epithelial ovarian cancer (EOC) cells (115). Downregulation of Sox2 reduces the tumorigenicity of cancer stem cells and regulates the expression of various cancer genes in lung cancer, including *c-MYC, WNT1, WNT2, and NOTCH1* (119). In addition, Sox2 downregulation in breast cancer and glioma cells results in decreased proliferation by cell cycle arrest (120).

## NANOG

Nanog is a homeodomain protein that is critical for mammalian development and specification of the ICM in the early embryo (16, 121). It forms dimers through its tryptophan-rich (WR) domain that is essential for ESCs’ self-renewal and pluripotency (122, 123). In addition, the dimer interacts with Kruppel-like zinc finger transcription factor Zfp281 (122) that functions as a transcriptional repressor for Nanog (124) while Patz1 (also a Kruppel-like zinc finger transcription factor) has the opposite effect as a transcriptional activator of Nanog (125). Nanog contains several phosphorylation sites at Ser/Thr-Pro motifs, which enable Nanog to be recognized and bound by the prolyl isomerase Pin1, leading to Nanog protein stabilization by preventing proteasome-mediated degradation (126). Phosphorylation and stabilization of Nanog by focal adhesion kinase (Fak) and protein kinase C*ϵ* (PKC*ϵ*) has also been associated with tumor development (127). More specifically, PKC*ϵ*-mediated phosphorylation translocates Nanog to the nucleus and activates miR-21 to promote breast tumor development and progression (128). Nanog is specifically expressed in ESCs, germ fibroblasts, and several tumor cell lines (129, 130). Knockdown of Nanog in gastric cancer cells reduced their proliferative and metastatic capacity, possibly as a result of increased apoptosis and cell cycle arrest (131). Similarly, Nanog was shown to exhibit anti-tumorigenic effects in glioblastoma (132), breast (133), and prostate (134) carcinoma cells. Furthermore, Nanog was found to promote chemoresistance and to increase cell migration and EMT (135, 136).

## Krüppel-Like Factor 4

Klf4 is a three-zinc finger TF with two nuclear localization signals (NLSs) discovered in 1996 and also known as gut-enriched krüppel-like factor (GKLF). KLF4 is highly expressed in skin and intestinal epithelial cells and is involved in the regulation of cellular proliferation and terminal differentiation of several different tissues such as intestinal, eye, and skin tissues. Moreover, Klf4 is a well-known key factor required to produce induced pluripotent stem cells (iPSCs) (137, 138), first discovered by Takahashi and Yamanaka (139). Dhaliwal et al. highlighted Klf4’s role to maintain pluripotency and prevent embryonic stem cell differentiation. It is maintained post-transcriptionally by Nanog and Sox2 where Sox2 co-expression enables KLF4 stability.

Klf4 is an important regulator of adipogenesis and together with Krox20 (early growth response protein 2) induces expression of C/EBP*β* through binding to C/EBP*β* promoter regions in conjunction with histone acetyltransferase p300. Prior induction of Klf4 *via* cAMP regulates C/EBP*β* expression, indicating a synergistic interaction. Conversely, knockdown of C/EBP*β* results in overexpression of Klf4 and Krox20 identifying C/EBP*β* as a downstream target (140). Klf4 knockdown is directly correlated with dysregulation of adipogenesis characterized by differentiation fat markers including peroxisome proliferator-activated receptor (PPAR*γ*) as it is mediated through C/EBP*β* (140). Ppar**γ**, in turn, regulates Klf4 expression *via* binding of the PPAR response element (PPRE) in its promoter making it a key transcription regulator of lipid metabolism (70). Ppar**γ** binding to Klf4 promoter induces the tumor suppression activity by affecting the complex pathways involving Klf4 in tumorigenesis as well as adipogenesis.

In cancer, particularly non-small cell lung cancer (NSCLC), Klf4 expression is downregulated in comparison to the surrounding normal tissues, indicative of a tumor suppressive function. Likewise, Klf4 has been found to act as a tumor suppressor in gastrointestinal cancer where it is associated with growth arrest through inhibition of G1/S cell cycle progression (71, 141). Klf4 has been reported to be a downstream target of methyltransferase like 3 (METTL3) using METTL3-depleted T24 bladder cancer cells. The cooperation of METTL3 with the reader protein YTH N6-Methyladenosine RNA Binding Protein 2 (YTHDF2) leads to the degradation of Klf4, which diminishes the tumor suppression activity of Klf4 and consequently induces cancer progression (72). Further, Klf4 negatively regulates serine/threonine kinase 33 (Stk33) by direct binding to its promoter, resulting in the inhibition of Stk33-induced EMT, a pivotal step in metastasis (73). In line with this, Klf4 expression has been correlated with inhibition of c-Jun N-terminal kinase (Jnk) which reportedly triggers EMT during cancer metastasis. In hepatocellular carcinoma (HCC), Klf4 was shown to regulate the expression of CD9/CD81, exosomal tetraspanin surface proteins that mediate cellular interaction and have been found to be involved in cancer (142). More specifically, CD9/CD81 were identified as transcriptional targets for Klf4 with a Klf4 binding site in their promoter regions. The expression of Klf4 was positively associated with the expression of CD9/CD81, and negatively affected downstream MAPK/JNK signaling, suggesting targeting Klf4-CD9/CD81-Jnk for future therapy. **Table 2** shows the expression levels of KLF4 involved in cancer processes.

**Table 2:** Expression of KLF4 and SALL1 in cancer suppression and carcinogenesis.

Klf4	Expression	Cancer Type	Reference
	Decreased	﻿Colorectal cancer	(70)
		Gastrointestinal cancer	(71)
		Bladder cancer	(72)
		Hepatocellular carcinoma (HCC)	(72)
	Decreased	Gastric cancer	(73)
	Increased	Breast cancer	(74)
	Decreased	Non-small-cell lung cancer (NSCLC) tissues	(75)
**Sall1**	**Activity**	**Cancer Type**	**Reference**
	Decreased	Breast cancer	(76)
		Myeloid leukemia (ML)	
		Cerebral glioma	(77)
	Decreased	Breast cancer	(76)
		Myeloid leukemia (ML)	
	Mutation	Rare human congenitalTownes–Brocks syndrome	(78, 79)

It is important to note that few studies have reported Klf4 to be a tumor promoting factor. For instance, Klf4 has been shown to mediate estrogen-induced mitogenic effects as it accumulates upon estrogen-induced downregulation of the ubiquitin protein ligase Von Hippel-Lindau (VHL) (74). Overexpression of KLF4 was shown to promote osteosarcoma cancer stem cells (143) and act as a tumor promoting gene in nasopharyngeal carcinoma (144). Finally, Klf4 promotes breast tumor development and is upregulated in 70% of breast tumors (71).

## Spalt-Like Transcription Factor 1

Sall1, together with Sall2, Sall3, and Sall4, forms the Sall family of zinc finger proteins containing cysteine–histidine residues (C2H2) (CX_2_–_4_CX_12_HX_2_–_6_H). Sall proteins are involved in organ development. Sall1 and Sall4 are specifically found to have an association with the rare human congenital Townes–Brocks syndrome that affects multiple organs (78, 79). Sall1 likely manifests this syndrome due to its role in kidney, heart, limbs, and central nervous system development (78). In this review, we focus on Sall1 as it is has been more frequently studied in the context of cancer as compared to the other Sall proteins.

The role of Sall1 in cell reprogramming was demonstrated through a Genome-Scale CRISPRa Screen (145), in which the expression of Sall1 was monitored individually and synergistically with Nanog. This study confirmed the capacity of Sall1 to reprogram primed epiblast stem cells (EpiSCs) and embryonic fibroblasts (MEFs) to iPSC, resulting in reprogramming the cell to ground state. In addition, Sall1 combined with Nanog maintained ESC state and regulated ESC reprogramming and differentiation. Concurrent overexpression of Nanog and Sall1 bestowed cells with the ability to form ESC colonies, whereas Sall1 alone was incapable of maintaining the ground state relative to Nanog’s ability. This work also showed that Sall1 and Nanog can delay differentiation of ESCs into EpiSCs *via* delayed upregulation of the differentiation markers Fgf5 (fibroblast growth factor 5) and Otx2 (orthodenticle homeobox 2).

In cancer, Sall1 has been found to be downregulated in breast cancer, glioblastoma (77), and myeloid leukemia, supporting its role as a tumor suppressor (76). In support of such a tumor suppressor role, Sall1 has been found to be a target of oncogenic miRNAs. For instance, Sall1 was found to be a potential target of the oncogenic miR-4286 in prostate cancer whereby miR-4286 knockdown abrogated Sall1’s ability to induce apoptosis and inhibit proliferation. Another study reported an inverse correlation, although not significant, between Sall1 and the oncogenic miR-181a-2 that is involved in microsatellite instability (146). **Table 2** highlights SAL1 expression in cancer modulation.

In addition to the zinc finger domains which are important for DNA binding, Sall1 is characterized by a rich glutamine domain responsible for dimerization. This domain comprises an N-terminal region with tumor-suppression and transcription repression activity, enabled by interaction with nucleosome-remodeling deacetylase complex (NuRD) (78), resulting in decreased tumor growth and proliferation, cell cycle arrest, and metastasis regression. Furthermore, overexpression of Sall1 negatively impacts cell cycle progression and proliferation through the suppression of *β*-catenin, antagonizing the Wnt/*β*-catenin signaling pathway accordingly by targeting *Wnt* downstream targets Cyclin D1 (Ccnd1) and c-Myc oncogene. In addition, Sall1 is affecting the progression of cancer through the upregulation of the epithelial marker E-cadherin and downregulation of the mesenchymal markers vimentin and N-cadherin, driving mesenchymal-to-epithelial transition (77).

## Gata TFs

The Gata family of TFs comprises zinc-finger DNA-binding proteins that control the development of diverse tissues, especially during hematopoiesis. They share conserved C2H2-type zinc-finger motifs (Cys-X2-C-X17-Cys-X2-Cys) that are involved in DNA-binding by recognizing the Gata element (A/TGATAA/G) (147). Based on expression pattern, they can be subdivided into two groups: Gata1, Gata2, and Gata3 forming the group of hematopoietic Gata factors, and Gata4, Gata5, and Gata6 grouped as endodermal Gata factors (148, 149). X-linked congenital anemia and thrombocytopenia have been linked to a point mutation within the N-terminal zinc finger of Gata1 that abolishes the interaction of Gata1 with the hematopoietic expressed transcription co-factor Fog1 (150). Gata3 plays an essential role in development and mammary gland function by maintaining the luminal cell lineage, and is expressed in differentiated luminal epithelial cells lining the breast ductal structures (151, 152). Gata3 gene deletion affects the mammary gland morphogenesis and in adults results in loss of luminal lineage (151, 153).

Since Gata proteins are heavily involved in regulating cell proliferation and survival of non-cancerous cells, it is evident how they can play a role in cancer. Altered expression or mutations of Gata factors are associated with a broad range of tumors including leukemia, colorectal, lung, breast, and brain tumors [Zhang et al., Rodriguez et al., Gao et al., Usary et al., Akiyama et al., Gong et al.]. Two mutations in the coding region (zinc finger domain) of Gata2 have been identified in a subset of human chronic myelogenous leukemia (CML). These mutations altered transactivation activity of Gata2 and its inhibitory effects on the activity of PU.1, a major regulator of myelopoiesis (154). In breast cancer, Gata3 expression is associated with invasive growth and poor prognosis (155). Its expression is maintained between primary and metastatic breast carcinoma and could potentially be used as a marker for metastatic breast carcinoma (156). Gata3 has also been suggested as a specific marker for urothelial carcinoma (157). Association of Gata3 with favorable clinicopathological parameters may indicate prognostic significance for Gata3 through its ability to promote luminal progenitor cells differentiation (158). Genomic analysis of breast cancer reveals high-frequency mutation in Gata3; however, most mutations were limited to a single allele, and expression of both mutated and wild-type alleles is approximately equivalent (159–161).

## Pax TFs

**Figure 3:**
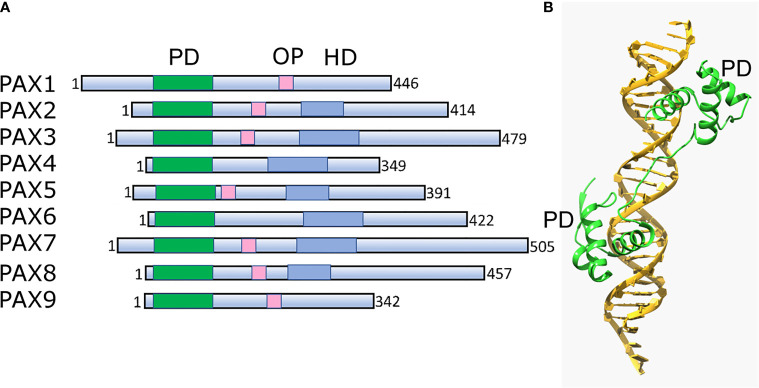
Structural architecture of Pax family and its binding to DNA. **(A)** Schematic drawing of conserved structural domains of Pax family members. The domains include the conserved paired domain (PD, green), the defining domain for this family. The Pax family members selectively contain other domains such as the homeodomain (HD, blue) and the octapeptide (OP, pink). **(B)** Ribbon diagram of Pax6 paired domain (PD)–DNA complex (PDBID: 6PAX). Crystal structure of the human Pax6 PD (primarily helix-turn-helix motif)-DNA complex reveals the region/subdomain involved in DNA binding.

Pax TFs are involved in multiple lineages to regulate cell fate during development and differentiation (162). They are sequence-specific DNA-binding proteins that are essential during early development and organogenesis (163). In general, Pax proteins are characterized by the presence of three conservative elements: two DNA-binding domains, the paired domain (PD) and homeodomain (HD), and the short octopeptide sequence (OP) located between PD and HD (**Figure 3A**). The paired domain, named after its first identification in the Drosophila gene *paired* (164), is the defining feature of this class of genes, while the OP and HD domains may be dispensable (**Figure 3B**).

The Pax family comprises nine members (Pax1–Pax9) in humans, subdivided into subgroups I–IV based on the presence, absence, or truncation of domains. Pax1 is expressed in cortical cells of the embryonic and adult thymus, where it participates in the maturation of thymocytes (165). It is often hypermethylated in cervical cancer and is a potential novel diagnostic biomarker (166). Pax2 binds to the promoter of a disintegrin and metalloproteinase domain-containing protein 10 (ADAM10), a metalloprotease that plays a crucial role in cancer progression and metastasis (167). It has been shown to regulate ADAM10 protein expression in renal cancer where it is expressed in 73% of cancer cells (168). Pax2 downregulation has been shown to lead to growth inhibition of cancer cells, and reactivation of Pax2 is also observed in clear cell renal cell carcinoma, a tumor type characterized by loss of VHL tumor suppressor function (169). Pax2 is also involved in cell proliferation and carcinogenesis in the endometrium, where it is activated by estrogen and tamoxifen, possibly due to cancer-linked hypomethylation of the Pax2 promoter (170). To date, very little information is available on Pax3 expression and function in cancer. In alveolar rhabdomyosarcoma, a pediatric soft tissue cancer related to the striated muscle lineage and characterized by the chromosomal translocations, chromosomal translocation events result in rearrangement of Pax3 and Pax7, juxtaposing these TFs with members of the fork head transcription factor family, and resulting in altered function from the chimeric gene product (171). Pax4 is involved in the differentiation and development of pancreatic islets. The high expression of Pax4 and the alternative splice variant Pax4v are critical in development of insulinoma through the upregulation of the anti-apoptotic gene bcl-xl (172, 173). Pax5 plays a vital role in all stages of B lymphocyte development (174). Reprogramming of mature B cells into pluripotent stem cells requires either ectopic expression of the myeloid transcription factor CCAAT/enhancer-binding-protein-alpha (C/EBPalpha) or Pax5 (175), in addition to core pluripotency TFs. Pax5 also mediates enhancer–promoter interactions and is able to alter genome topology, even in untranscribed regions (176). Pax6 regulates the neuroectoderm formation from ESCs, neural stem cell proliferation, neural stem cell self-renewal, neurogenesis and is critical for the development of the central nervous system (177, 178). Pax6 forms a complex with Sox2 on the lens-specific enhancer elements known as delta-crystallin minimal enhancer (DC5). Pax6 alone shows a poor binding on DC5; however, it cooperatively forms a stable ternary complex with Sox2 to the DC5 cis element, correlating with the enhancer activation required for eye development (95, 179). Pax6 is overexpressed in pancreatic carcinoma cell lines and promotes cancer progression by directly binding and activating the MET tyrosine kinase receptor (180). In contrast, Pax6 suppresses glioblastoma cell growth by downregulating the expression of the gene encoding vascular endothelial growth factor A (VEGFA) (181). Pax7 plays an important role in skeletal muscle formation (182). PAX8 is abundantly expressed in renal tissues and is a nephric-lineage TF required for the formation of the kidney (183). Pax8 expression is also frequently observed in renal, bladder, ovarian, and thyroid cancer cells. Silencing of Pax8 leads to a reduction in the expression of E2F1 and proteasome-dependent destabilization of the tumor suppressor retinoblastoma protein (RB) (184). Pax8 is also involved in telomerase regulation, telomerase reverse transcriptase and telomerase RNA component, in glioma (185). In thyroid carcinoma, Pax8 exists as a gene fusion with peroxisome proliferator activated receptor gamma (Pax8/PPARG gene fusion), resulting in an oncogenic Pax8–PPARγ fusion protein (186). Similar to Pax1, Pax9 is expressed in embryonic and adult thymus (165). In lung cancer, amplification of Pax9 promotes cell proliferation of lung cancer cells (187). Conversely, inhibition of Pax9 in oral squamous cell carcinoma triggers the induction of apoptosis corroborating its critical role in cell growth and survival, and thus disrupting the function could be a potential avenue for cancer treatment (188).

## PPARγ TFs

Ppar**γ** TFs, together with C/EBPs and the basic helix–loop–helix family (ADD1/SREBP1c), play a crucial role in adipogenesis, a process that involves cellular differentiation and morphological changes in cell size and lipid content (189–191). Ppar**γ** is a member of the nuclear hormone receptor superfamily and requires heterodimerization with retinoid X receptor or Rxr to bind DNA and be transcriptionally active (192, 193). It can be present as two protein isoforms through alternate promoters and splicing whereby Ppar**γ** 2 the dominant isoform is in fat cells with an extra 30 amino acids at the N-terminus compared to Ppar**γ**1 (192).

Given its prominent role in adipogenesis, it is not surprising that increased expression of Ppar**γ**/Rxr has been found in liposarcomas that were triggered to undergo terminal differentiation *in vitro* by thiazolidinediones or TZDs (class of antidiabetic drugs) and Rxr-specific retinoids (194). These results suggest that these compounds may be useful drugs to differentiate liposarcomas through maximal activation of the Ppar**γ** pathway (194). Additionally, thiazolidinedione could be used as a non-toxic alternative to conventional chemotherapy for the treatment of locally advanced liposarcoma (194). Nevertheless, TZDs have shown only modest therapeutic benefit in clinical trials over the past 15 years (195). Factors affecting drug efficacy could include compound-specific effects, the necessity of Ppar**γ** activation or other targets, the tumor stage at the time of drug exposure, the age of the patient, and finally the influence of TZDs on cancer cell paracrine activity (195–197). In addition, Ppar**γ** can inhibit *β*-catenin that activates Pdk1 and Cyclin D1 (198) and upregulates Myc (199).

**Figure 4:**
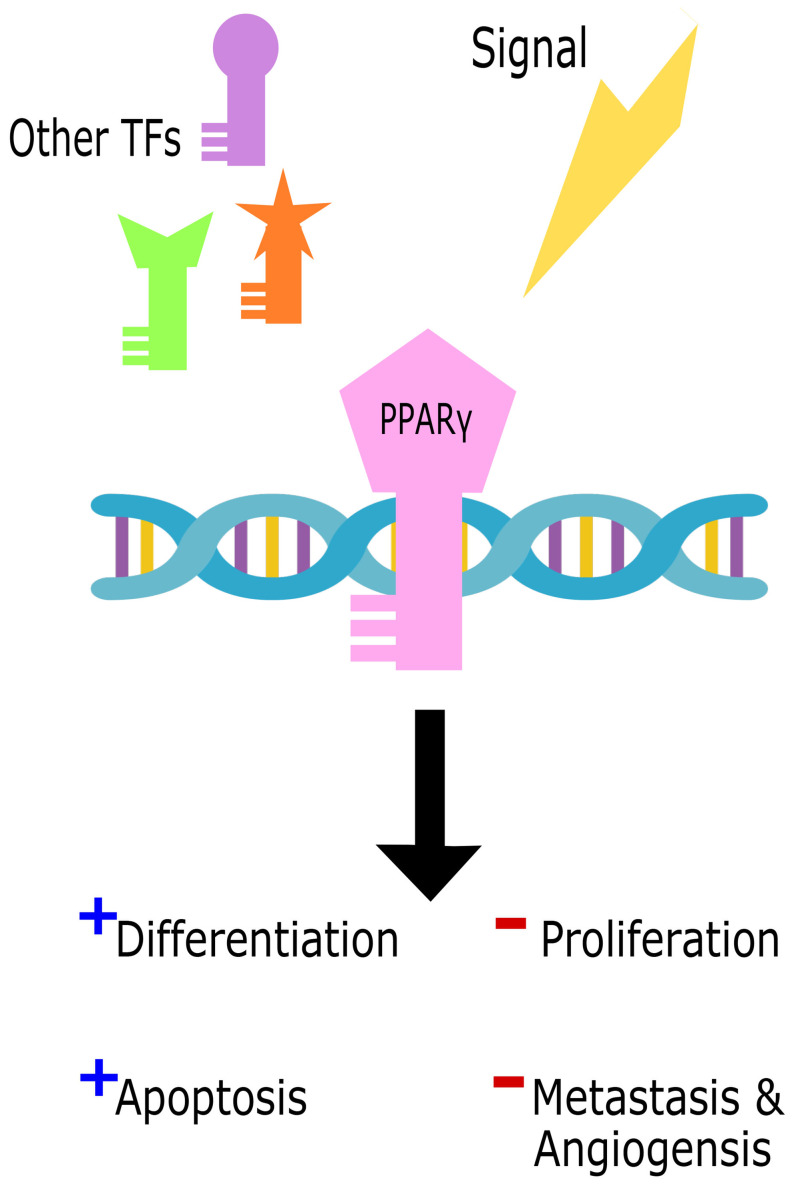
The dual role of Ppar*γ* in the cell. Cell signals in parallel with other transcription factors (TFs) trigger Ppar*γ* binding to DNA to initiate either tumor suppressive promoting functions.

On the other hand, several studies revealed a pro-tumorigenic role for Ppar**γ** in urinary bladder cancer, promoting tumorigenesis, metastasis, and angiogenesis (200–202) through several pathways including adipose differentiation and cell cycle arrest. The pro-tumorigenic function of Ppar**γ** can be induced by increased inflammation through the upregulation of IL-6/STAT3 (203), Cox2, and PGE2 (204). Other pathways also give rise to cancers due to Ppar**γ** mutations with partial loss of function or chimeric mutations such as in colon cancer (205), prostate cancer (206), and thyroid tumors (207, 208) where Ppar**γ** levels were associated with tumor grade and invasive ability. **Figure 4** illustrates the dual role of Ppar**γ** within the cell.

Thus, Ppar**γ** exhibits a context-dependent pro- or anti-tumorigenic behavior, which needs to be carefully considered prior to therapeutic intervention.

## Pok Family

The POK transcription repressors (also named POZ-ZF transcription factors) are a major family of transcription factors which have a dual role in development and cancer. Apart from their involvement in several fundamental biological processes, they also participate in hematopoiesis, adipogenesis, chondrogenesis, DNA repair, development of oligodendrocytes, osteoclast, and unfolded protein response (209). The POK family (present in approximately 43 human genes) is composed of one or more C-terminal C_2_H_2_ *Krüppel*-type zinc finger domains, which are DNA binding domains, coupled with an N-terminal POZ/BTB (broad-complex, tramtrack, and Bric a brac) domain used for protein–protein interactions, allowing recruitment of corepressor complexes. The hinge region between the POZ/BTB and ZF domains and the C-terminal end of the ZF domain are often targeted for post-translational modification and regulation (210). The members of this family enable their regulation by binding of the zing finger domain in their target genes followed by recruitment of various cofactors (NCoR, SMRT, Sin3a) through the N-terminal domain for chromatin remodeling and transcriptional silencing or activation (211).

This family includes Bcl-6, PLZF, PATZ1 (also named MAZR), Kaiso, and many others (212, 213). Members include PLZF (promyelocytic leukemia zinc finger), which is involved in limb and skeleton development (214), regulates spermatogenesis (215) as well as natural killer T-cell (NKT) development (216). PLZF is linked to tumor suppression *via* its transcriptional repression of the c-myc oncogene (217). PATZ1/MAZR (AT-hook containing zinger finger protein 1) is similar and has been implicated in spermatogenesis (218), pluripotency maintenance (125), and in different developmental processes, including neural development (219) and T cell differentiation (220). However PATZ1/MAZR has also been described to act as an oncogene or tumor suppressor in experimental tumors and human cancer (221). Bcl-6 (B cell lymphoma 6) is critical in B cell development and is also dysregulated in B cell lymphoma (222, 223). Kaiso is involved in intestinal cell fate by regulating Notch signaling (224) and promotes EMT in prostate cancer by regulating miR-200c (225).

Thus this family of proteins carries out key steps in developmental pathways, and dysfunction can lead to carcinogenesis through several pathways involved in cell fate decisions, cell cycle control and apoptosis.

## Targeting Transcription Factors in Cancer: Potential and Challenges

A plethora of evidence has identified CSC transcription factors that can drive tumorigenesis. CSCs additionally display resistance to chemotherapy (226) and radiotherapy (227, 228), thus rendering them capable of repopulating tumors in pre-treated relapsing patients. As such, strategies to target CSCs are lucrative to improve treatment response and disease-free survival. However, until recently, this class of proteins were considered “undruggable” (229). Firstly, transcription factors’ function broadly as master regulators in an immense repertoire of signaling pathways regulating normal tissue homeostasis, thus highlighting a need for targeted inhibition in cancer cells. Secondly, the lack of enzymatic activity and hence binding sites has rendered designing small molecule inhibitors challenging. In addition, the redundancy and functional compensation of transcription factors may limit the efficacy of single agent therapy. Consequently, the majority of currently available CSC TF modulators are non-selective or target upstream molecules (**Table 3**). For example, fursultiamine (thiamine tetrahydrofurfuryl disulfide, TTFD), a derivative of vitamin B, has been reported to suppress the expression of several CSC TFs including Oct4, Sox2, and Nanog resulting in reduced stem cell properties in esophageal carcinoma spheroids and mice xenografts (237). In addition, TTFD treatment also improved the response to concurrent chemoradiotherapy in the same mouse model. This combination modality has been investigated in a phase II clinical trial (NCT02423811) of esophageal squamous cell carcinoma, and the results are still pending. Similarly, a synthetic compound PT‐262 (7‐chloro‐6‐piperidin‐1‐yl‐quinoline‐5, 8‐dione) has been shown to inhibit the expression of Oct4 and Nanog, concurrent to suppressing the growth of lung tumor xenografts in mice (238). Furthermore, few drugs have been identified that affect upstream regulators of CSC TFs (**Table 3**). MLN4924, also known as pevonedistat, is a neddylation inhibitor that induces the accumulation of MSX2, a known transcription repressor of Sox2. MLN4924-mediated Sox2 downregulation has been shown to suppress stem cell properties and to exert broad anti-cancer effects both in *in vitro* and *in vivo* models (239, 240). Several phase I/II clinical trials are investigating single agent pevonedistat and its combination with standard chemotherapy in mesothelioma (NCT03319537), acute myeloid leukemia (AML) (NCT03009240, NCT00911066, NCT03330821, NCT03009240, NCT03459859, NCT03772925), acute lymphoblastic leukemia (NCT03349281), chronic lymphocytic leukemia (NCT03479268), relapsed or refractory lymphoma or multiple myeloma (NCT00722488, NCT03323034, NCT03770260), melanoma (NCT01011530) and non-hematologic malignancies (NCT00677170, NCT01862328) such as advanced non-small cell lung cancer (NSCLC, NCT03965689, NCT03228186) and intrahepatic cholangiocarcinoma (NCT04175912). In line with promising observations from pre-clinical studies (241), numerous phase I/II clinical trials are assessing the combination of pevonedistat with 5-azacytadine in newly diagnosed or relapsed/refractory AML or myelodysplastic syndrome (NCT03813147, NCT02782468, NCT04172844, NCT03238248, NCT02610777). This combination has now progressed into phase III trials in newly diagnosed AML not eligible for intensive chemotherapy (NCT04090736) and higher-risk myelodysplastic syndromes, chronic myelomonocytic leukemia, or low-blast AML (NCT03268954). Although single agent pevonedistat indicated modest clinical benefit (242–246), a combination of pevonedistat treatment with carboplatin and paclitaxel in advanced solid tumors (NCT01862328, 35% objective response rate) (247) or with 5-azacytadine in treatment-naïve AML patients (NCT01814826, >50% ORR) (248) showed promising anti-tumor activity. Both these studies did not indicate any additional toxicity to those elicited by chemotherapy or 5-azacytadine treatment alone. However, transient elevations in liver function tests were dose limiting for pevonedistat treatment. Likewise, modulators of calcium signaling such as thapsigargin, a Sarco/Endoplasmic Reticulum Ca(2+)-ATPases (SERCA) inhibitor induce a rise in cytosolic Ca2+ levels, which activates Akt-mediated phosphorylation and subsequently inhibits the oncogenic fusion transcription factor Pax3-FoxO1 (249). Accordingly, thapsigargin treatment suppresses the growth of Pax3-FoxO1 expressing alveolar rhabdomyosarcoma cell lines and xenografts.

**Table 3:** Inhibitors of cancer stem cell transcription factors.

Candidate drug	Target	Pre-clinical studies		Clinical studies		Reference
		Cancer type	Effect	Cancer types	Effect	
Fursultiamine (thiamine tetrahydrofurfuryl disulfide, TTFD)	Non-selective	Esophageal squamous cell carcinoma	• Suppressed OCT-4, SOX-2, NANOG expression in spheroids • Reduced CSC phenotype in spheroids • Improved xenograft response to CCRT	• Esophageal squamous cell carcinoma *(NCT02423811)*	Results pending	(230)
PT‐262 (7-chloro‐6‐piperidin‐1‐yl‐quinoline‐5, 8‐dione)	Non-selective	Lung cancer	• Inhibited OCT-4 and NANOG expression • Inhibited anchorage-independent ability and tumor growth in mice	NA	NA	(231)
MLN4924/Pevonedistat	NAE inhibitor	Breast cancer AML	• Depleted SOX-2 *via* targeting the FBXW2-MSX2 axis • Suppressed CSC properties • Sensitized breast cancer cells to tamoxifen • Combination with 5-AZA increased DNA-damage, cell death and complete xenograft tumor regression	• Mesothelioma *(NCT03319537)* • AML and MDS *(NCT03009240, NCT00911066, NCT03330821, NCT03009240, NCT03459859, NCT03772925, NCT03813147, NCT02782468, NCT04172844, NCT03238248, NCT02610777, NCT04090736, NCT03268954)* • ALL *(NCT03349281)*, CLL *(NCT03479268)* • Lymphoma or multiple myeloma *(NCT00722488, NCT03323034, NCT03770260)* • Melanoma *(NCT01011530)* • Non-hematologic malignancies *(NCT00677170, NCT01862328, NCT03965689, NCT03228186, NCT04175912)*	• Combination with carboplatin and paclitaxel *(NCT01862328)* shows 35% ORR in advanced solid tumors • Combination with 5-AZA *(NCT01814826)* shows >50% ORR in treatment-naïve AML patients	(41–45, 232–236)
Thapsigargin	SERCA inhibitor	Alveolar rhabdomyosarcoma	• Inhibits the fusion PAX3-FOXO1 TF Suppressed cell line and xenograft growth	NA	NA	(46)
EG1	PAX2 DNA binding domain	Renal and Ovarian cancer	• Inhibits PAX2 activity • Anti-proliferative effects in cell lines	NA	NA	(47)
NSC140905 (2-(1,3-benzodioxol-5-ylmethyl)butanedioic acid)	GATA4 DNA binding domain	Meningioma cancer	• Inhibits GATA4 activity • Decreased cancer cell viability • No effect on normal meningeal cells	NA	NA	(48, 49)
Efatutazone/CS-7017	PPAR-γ agonist	Anaplastic thyroid carcinoma	• Increased cancer cell death • Inhibited proliferation • Suppressed cancer cell motility	• Metastatic or locally advanced NSCLC (NCT01199068, NCT01101334, NCT01199055, NCT00806286) • Metastatic colorectal cancer (NCT00986440 NCT00967616) • Anaplastic thyroid cancer (NCT02152137)	• Partial responses and stable disease in various solid tumors	(22, 50–56)
		EGFR-TKI-resistant lung adenocarcinoma			
Pyrrothiogatain	GATA DNA binding domain	Th2 cells	• Inhibits GATA3 activity • Inhibits GATA3/SOX-4 interaction	NA	NA	(57)

5-AZA,5-azacytidine; ALL, acute lymphoblastic leukemia; AML, acute myeloid leukemia; CCRT, concurrent chemo radiotherapy; CLL, chronic lymphocytic leukemia; EGFR-TKI, epidermal growth factor receptor-tyrosine kinase inhibitor; MDS, myelodysplatic syndrome; NA, not available; NAE, NEDD8-activating enzyme; NSCLC, non-small cell lung cancer; PPARγ, peroxisome proliferator-activated receptor gamma; SERCA, sarco/endoplasmic reticulum Ca(2+)-ATPase; TF, transcription factor; Th2, T helper cells.

Due to challenges in designing selective inhibitors for transcription factors, potential strategies have focused on disrupting their binding to DNA (**Table 3**). In this regard, a compound termed EG1 was reported to target the DNA binding domain of Pax2, thereby blocking its transcriptional activity. EG1 treatment has demonstrated anti-proliferative effects in Pax2 expressing renal and ovarian cancer cell lines; however, its efficacy *in vivo* has not yet been reported (250). Similarly, pyrrothiogatain has been identified as a DNA-binding inhibitor of the Gata family, particularly of Gata2–Gata5, in addition to inhibiting the interaction of Sox4 and Gata3 (251). However, its effect on cancer cells remains to be investigated. Furthermore, the synthetic derivative of succinic acid NSC140905 [2-(1,3-benzodioxol-5-ylmethyl)butanedioic acid] was reported to bind to the DNA-binding domain of Gata4, thus blocking its transcriptional activity (252). Of note, treatment of meningioma cancer cells with NSC140905 decreased cancer cell viability but did not affect normal human meningeal cells *in vitro* (253). The potential of Sox decoy molecules, which target their DNA binding activity, has also been demonstrated to inhibit Sox18-induced genes in the COS-7 cell line (254). These decoys have been designed to resist nuclease digestion, degradation, and thermal denaturation *in vitro* but remain to be investigated in pre-clinical cancer models.

As transcriptions factors typically interact with numerous proteins downstream of signaling cascades, targeting such partner proteins may potentially affect their transcriptional activity. For instance, FoxA1 interacts with the cyclin-dependent kinase 1 (Cdk1) cell cycle regulator in certain types of breast cancer cells (255). Additionally, *in silico* analyses has indicated that Cdk-mediated phosphorylation of FoxA1 may potentially regulate FoxA1 binding to DNA. Consequently, treating these cell lines with Cdk inhibitors suppresses FoxA1 binding to DNA (255). Theoretically, this may also negatively affect cancer cell proliferation and hence, requires further investigation. In contrast, strategies targeting the CSC TF Ppar*γ* focus on activating this tumor suppressor to mitigate oncogenesis (256). Ppar*γ* agonists, particularly the thiazolidinedione class of ligands (troglitazone, rosiglitazone, and pioglitazone), have been commonly used as anti-diabetic drugs. Although these drugs have shown pre-clinical anti-proliferative effects in numerous cancer types (257–259), their administration in clinical trials has indicated limited efficacy (260, 261). A novel, third generation thiazolidinedione, efatutazone or CS-7017, is significantly more potent than its predecessors in inducing Ppar response element activation and anti-tumor activity, and thus might exhibit a higher efficacy in clinical setting (262, 263). Pre-clinical studies have shown that efatutazone in combination with chemotherapy can increase cancer cell death, inhibit proliferation, and suppress cancer cell motility of particularly epidermal growth factor receptor-tyrosine kinase inhibitor (Egfr-Tki)-resistant lung adenocarcinoma cells (230–232, 264). Clinically, single agent efatutazone therapy and efatutazone therapy in combination with chemotherapy have induced partial responses and stable disease in various solid tumors (20, 233–235). Although efatutazone treatment demonstrated acceptable tolerability, peripheral edema was commonly observed as an adverse effect, with patients often requiring diuretics. Furthermore, numerous ongoing phase I and II clinical trials are assessing the synergistic efficacy of efatutazone with the Egfr-Tki Erlotinib (NCT01199068, NCT01101334) or carboplatin/paclitaxel (NCT01199055, NCT00806286) in metastatic or locally advanced NSCLC, with irinotecan, leucovorin, and 5-fluorouracil chemotherapy in metastatic colorectal cancer (NCT00986440 NCT00967616) and with paclitaxel in anaplastic thyroid cancer (NCT02152137).

Emerging technologies in high-throughput screening are shifting the “undruggable” paradigm towards identifying selective modulators of cancer-associated transcription factor activity (236). Moreover, efforts towards designing targeted delivery of small molecules, including synthetic compounds, short-interfering RNA or Clustered Regularly Interspaced Short Palindromic Repeats (CRISPR) genome editing tools, could transform cancer treatment to specifically target transcription factors and their mutant alleles in tumor cells with minimal off-target effects. Finally, the functional redundancy of CSC TFs could be overcome by combining TF modulators with or without standard cancer treatment, which has already been indicated by the improved efficacy of clinical trials combining CSC TF modulators with chemotherapy.

## Conclusion

This review attempts to summarize the choices of regulated cell fate decisions *versus* dysfunction leading to cancer meted out by several transcription factors. Key TFs were chosen which are known to have important cell fate roles as well as dysfunction during carcinogenesis. This review covers early players in stem cell development such as Oct4 and Sox2 as well as other TFs in early differentiation events such as Gata, Pax, and Ppar**γ**. Different criteria including expression levels and mutations in critical functional domains are described and how they exert their effects for several different cancers. Finally, this review describes the potential for drugging different cancers using various compounds which specifically could mitigate the “stemness” of cancers. Understanding how the TFs conspire for normal cellular development *versus* malignant outcomes will be critical in developing better selective ligands that can target cancer with fewer side effects in the future.

## Author Contributions

PK had the principal idea to combine applications of transcription factors with stem cell development and tumorigenesis for the review. ZI, AA, and ME also added many specifics about stem cells and pluripotency details as well as tumorigenesis and metastasis due to the relevant transcription factors. AN and JD were the principal contributors for the clinical relevance to cancer as well as other cancer specifics. All authors contributed to the article and approved the submitted version.

## Funding

This work was supported by two intramural grants (IGP) from the Qatar Biomedical Research Institute, awarded to PK and JD, respectively. In addition work was also supported by a QNRF NPRP grant NPRP11S-0120-180346 to PK.

## Conflict of Interest

The authors declare that the research was conducted in the absence of any commercial or financial relationships that could be construed as a potential conflict of interest.
